# PCPDTBT Conjugated Polymer Nanoparticles for Photothermal Inactivation of Multidrug-Resistant *mcr‑1*-Positive *Escherichia coli*

**DOI:** 10.1021/acsomega.5c04993

**Published:** 2025-08-25

**Authors:** Cynthia S. A. Caires, Rafael C. Nascimento, Leandro O. Araujo, Laís F. Aguilera, Samuel L. Oliveira, Anderson R. L. Caires

**Affiliations:** Instituto de Física, 54534 Universidade Federal de Mato Grosso do Sul, CP 549, Campo Grande, 79070-900 Mato Grosso do Sul, Brazil

## Abstract

Antimicrobial resistance (AMR) poses a global health challenge, threatening the effectiveness of the current treatment of bacterial infections. The emergence of plasmid-mediated resistance, notably the *mcr-1* gene in *Escherichia coli* (*E. coli*), further complicates therapeutic options by conferring resistance to colistin, a last-resort antibiotic. This study explores the potential of conjugated polymer nanoparticles made of poly­[2,6-(4,4-bis­(2-ethylhexyl)-4*H*-cyclopenta­[2,1-*b*;3,4-*b*′]­dithiophene)-*alt*-4,7­(2,1,3-benzothiadiazole)] (PCPDTBT NPs) for antimicrobial photothermal therapy (PPT) against multidrug-resistant, *mcr-1*-positive *E. coli* strains. PCPDTBT NPs were synthesized through nanoprecipitation and characterized for their photothermal response upon near-infrared (NIR) laser irradiation at 806 nm (1.13 W). The nanoparticles exhibited efficient absorption in the NIR range and generated substantial photothermal heating sufficient for bacterial inactivation. PCPDTBT NPs maintained their photothermal performance without degradation across multiple irradiation cycles. Photoinactivation assays confirmed PCPDTBT NPs’ (17 mg L^–1^) ability to significantly reduce bacterial viability, particularly against *mcr-1-*positive *E. coli*. Scanning electron microscopy (SEM) images confirmed pronounced damage to bacterial cells following photothermal treatment. Overall, PCPDTBT NPs are highly promising as standalone agents for PPT against antibiotic-resistant pathogens, indicating their potential for future therapeutic strategies.

## Graphical abstract

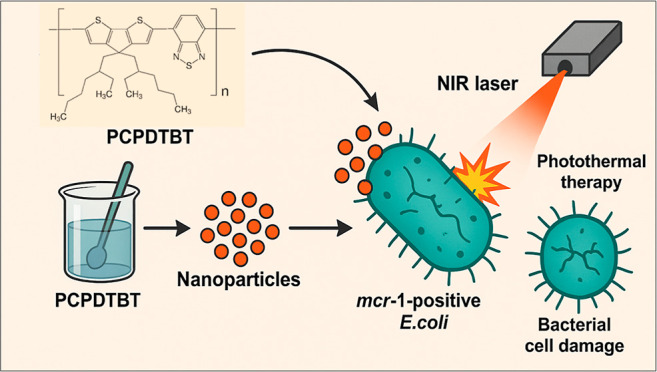

## 1 Introduction

The World Health Organization (WHO) has identified antimicrobial resistance (AMR) as one of the top 10 global public health threats.^1^ AMR in bacterial pathogens is a growing pandemic that threatens to render current treatments for microbial infections ineffective, bringing back a preantibiotic era. In 2019, approximately 1.27 million people worldwide died from infections directly caused by antibiotic-resistant bacteria.^2,3^ Without innovative interventions, global deaths associated with AMR are projected to reach 10 million annually by 2050.^4^

The widespread prevalence of AMR is partly due to the horizontal transfer of antibiotic resistance genes (ARGs), often facilitated by plasmids.^5^ One of these plasmid-mediated resistance genes, the *mcr-1* gene, was first reported in China in 2016. This gene allows *Escherichia coli* (*E. coli*) to resist colistin antibiotics in animals, food, and humans.^6^

Colistin, a broad-spectrum polymyxin antibiotic, is a last-resort treatment for infections caused by Gram-negative bacteria resistant to multiple drugs.^7^ Ten variants of the *mcr* gene have been identified.^8^ Despite documented genetic variations of *mcr-1* indicating ongoing evolution, these variants have not spread globally like the original *mcr-1* strain.^7,9^ The emergence of *mcr-1*-mediated colistin resistance in *E. coli* has imposed innovative strategies to restore antibiotic efficacy.^10^ Recent studies highlight the potential of synergistic drug combinations, such as tetrandrine with colistin, which disrupts bacterial membranes and inhibits *mcr-1* activity by binding critical residues in its active site.^11^ Similarly, the natural compound Rhein enhances colistin sensitivity by damaging bacterial membranes, depleting proton motive force, and downregulating *mcr-1* expression.^12^ Antimicrobial peptides like MSI-1 further combat resistance by suppressing *mcr-1* production, modifying the outer membrane vesicle biogenesis, and boosting immune responses through caspase-11-dependent pyroptosis.^13^ These approaches not only restore colistin’s bactericidal effects but also mitigate immune evasion and delay resistance development. Differently, photodynamic inactivation has emerged as a promising strategy to combat *mcr-1*-positive *E. coli*, leveraging the generation of reactive oxygen species (ROS) and singlet oxygen (^1^O_2_) to induce oxidative damage in bacterial cells.^14^ This approach is particularly advantageous due to its multitarget mechanism, which minimizes the likelihood of resistance development compared to conventional antibiotics. For instance, conjugated polymer nanoparticles (CPNs) have demonstrated significant efficacy in photoinactivating *mcr-1*-positive *E. coli* under white light irradiation.^15^

In this context, antimicrobial photothermal therapy (aPTT) is emerging as a promising strategy to eliminate bacterial cells through hyperthermia.^16,17^ Zhao et al. demonstrated that photothermal therapy (PTT) offers several advantages, including being noninvasive, having low toxicity, requiring simple operation, possessing broad-spectrum antibacterial ability, and reducing the likelihood of developing drug resistance. These factors make this therapy unique for combating microbial infections.^18^

Conjugated polymer nanoparticles (CPNs) are multifunctional nanoscale materials that exhibit versatile applications across various technological fields, drawing attention for their use in photothermal and photodynamic strategies to combat multidrug-resistant bacteria.^19–23^ The photothermal approach can be achieved by combining the use of CPNs under near-infrared (NIR) illumination,^24^ in which CPNs act as photothermal agents (PTAs) because they efficiently convert NIR light into heat, resulting in heat-induced bacterial death promoted by localized hyperthermia.^25,26^ This method offers a novel, noninvasive approach to tackling antibiotic resistance, reducing the dependence on conventional treatments,^22,25^ whose effectiveness has already been demonstrated in vitro and in vivo.^18,27^

Poly­[2,6-(4,4-bis­(2-ethylhexyl)-4*H*-cyclopenta­[2,1-*b*;3,4-*b*′]­dithiophene)-*alt*-4,7­(2,1,3-benzothiadiazole)], also known as PCPDTBT, is a conjugated polymer with luminescent properties that has also been studied as a PTA under NIR light, acting as a theranostic agent for photothermal and imaging applications.^26,28,29^ MacNeill et al. (2013) demonstrated that PCPDTBT nanoparticles can effectively generate localized heat upon NIR irradiation, enabling thermal ablation of cancer cells in vitro.^30^ Subsequent study has highlighted how molecular ordering within nanoparticle assemblies significantly influences their photothermal performance.^31^ Additionally, PCPDTBT-based systems have been integrated into multifunctional nanoplatforms for synergistic therapeutic strategies. For instance, Zhou et al. (2022) developed chemiluminescent nanoprobes incorporating PCPDTBT for deep-tissue bacterial imaging and combined photothermal-nitric oxide therapy.^32^ More recently, Wu et al. (2025) demonstrated that NIR-responsive polymer dots containing PCPDTBT can enhance the synergistic effect of photothermal therapies in vitro.^29^ Together, these studies underscore PCPDTBT’s versatility and efficacy as a core photothermal material in biomedical applications, with significant potential for in vivo applications due to its established biocompatibility and biodegradability.^33,34^ However, long-term in vivo studies of clearance mechanisms and immune responses remain an area for further investigation to fully validate its clinical translational potential.

Although PCPDTBT has been investigated as a PTA, its photothermal action against multidrug-resistant bacteria remains unexplored. The present study shows that PCPDTBT NPs produced by the nanoprecipitation method efficiently photoinactivate the *mcr-1* *E. coli* strain after a few minutes of NIR irradiation.

## 2 Materials and Methods

### 2.1 Nanoparticle Synthesis

Conjugated polymer poly­[2,6-(4,4-bis­(2-ethylhexyl)-4*H*-cyclopenta­[2,1-*b*;3,4-*b*′]­dithiophene)-*alt*-4,7­(2,1,3-benzothiadiazole)] (PCPDTBT) from Sigma-Aldrich was used to produce nanoparticles by nanoprecipitation.^15^ PCPDTBT was added dropwise to tetrahydrofuran (THF) at 0.23 mg mL^–1^ to 10 mL of an aqueous solution of polysorbate 20 (Tween 20, Quimesp Química, Brazil) at 1.2 mg mL^–1^ under slow stirring. After the mixture was stirred for 12 h, the evaporated volumes of water and THF were refilled with the surfactant solution to maintain the nanosuspension volume at 10 mL (Figure 1a). The final concentration of PCPDTBT NPs in the stock solution was 55.3 mg L ^–1^.

**Figure 1:**
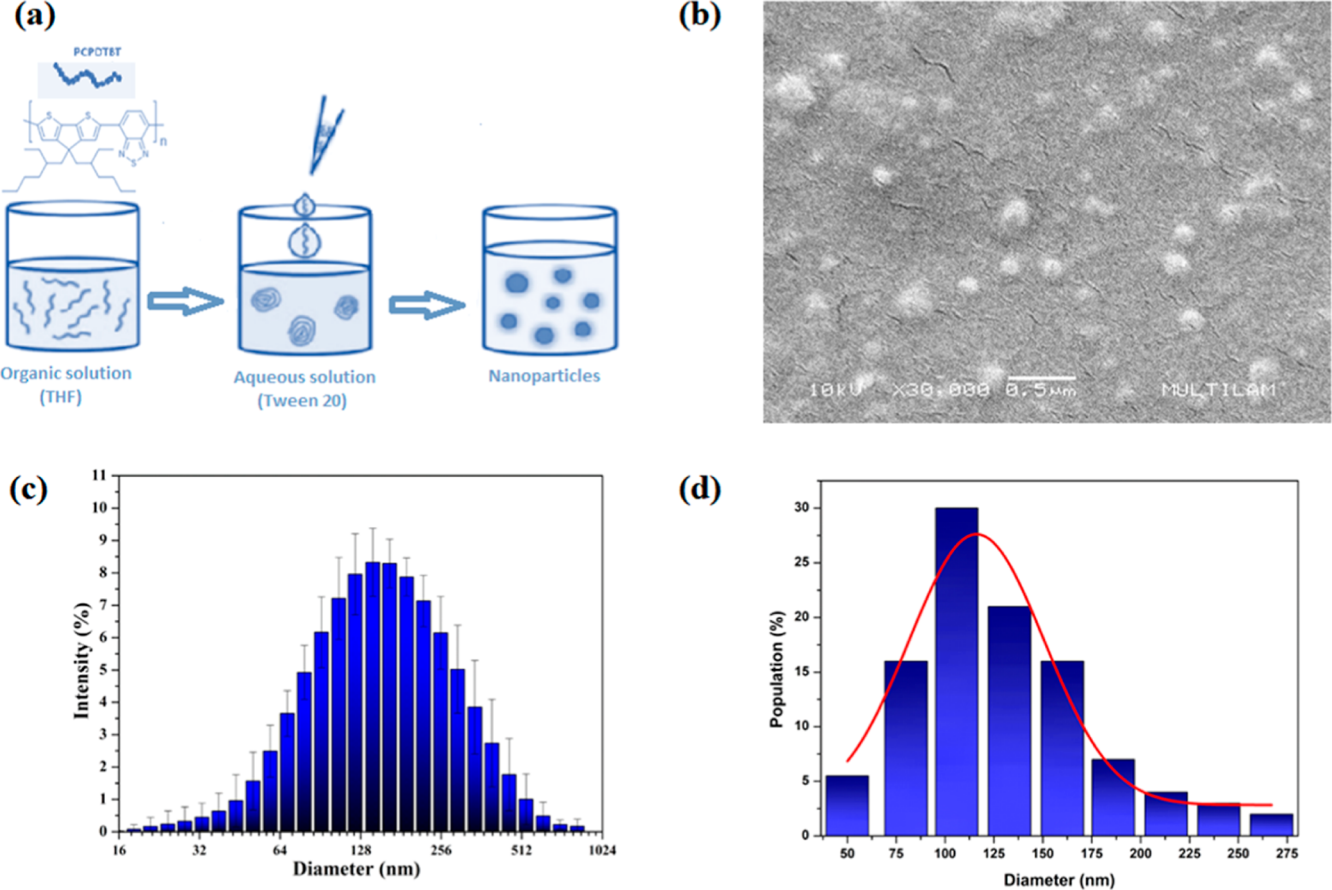
(a) Schematic representation of the nanoprecipitation process; (b) scanning electron microscopy (SEM) image of PCPDTBT nanoparticles; (c) hydrodynamic diameter distribution of PCPDTBT NPs; (d) particle diameter distribution determined from SEM image analysis.

### 2.2 Characterization

#### 2.2.1 UV–vis–NIR Absorption

UV–vis–NIR spectroscopy was carried out using a Shimadzu UV-2600i spectrophotometer equipped with an ISR-2600 Plus integrating sphere accessory, which collected the absorption spectrum in the range of 300–900 nm.

#### 2.2.2 Size and Morphology

The size and morphology of PCPDTBT NPs were analyzed by using scanning electron microscopy (SEM) (JEOL model JSM-6380LV) at an operating voltage of 10 kV. A solution of PCPDTBT NPs was applied onto a 1 cm × 1 cm glass substrate and allowed to dry overnight at room temperature. The dried sample was sputter-coated with gold and attached to SEM holders with carbon. ImageJ software was used to analyze SEM images to determine the particle diameter distribution and morphology. The average size was calculated by assuming a spherical shape for 100 particles. The hydrodynamic size, polydispersity index, and zeta potential were measured using dynamic light scattering (DLS) with a Zetasizer NanoZS device from Malvern Instruments Ltd., UK. The mean hydrodynamic diameter of PCPDTBT NPs was measured at 25 °C immediately after diluting to a concentration of 4.9 μg mL^–1^. NaCl was added to the PCPDTBT NP solutions to achieve a final concentration of 9 mM for the zeta potential measurements.

#### 2.2.3 Photothermal Stability

The photothermal stability of the PCPDTBT NPs was evaluated using an FLIR C5 thermographic camera. A solution of PCPDTBT NPs (17.0 mg L^–1^) was irradiated with an 806 nm laser (1.13 W, B&W Tek Inc.), and temperatures were recorded at 1 min intervals across eight irradiation cycles. Tests were conducted as a function of time, laser power (0.22, 0.52, 0.81, and 1.13 W), and nanoparticle concentrations (0, 4.25, 8.50, and 17.0 mg L^–1^).

### 2.3 Bacterial Strains

The experiments employed a nonmultidrug-resistant *E. coli* strain (ATCC 25922) and a multidrug-resistant *mcr-1* positive strain (CCBH 23595). These strains were preserved in Muller Hinton Broth with 20% v v^–1^ glycerol in a freezer. For bacterial suspension preparation, 40 μL of each strain was added to 2 mL of Brain Heart Infusion (BHI), and the mixture was incubated at 37 °C for 24 h. A 0.5 McFarland turbidity standard was used to standardize the bacterial concentration. Turbidity was evaluated using a UV–vis spectrophotometer with a deuterium-tungsten light source (DH-2000, Ocean Optics) to measure the absorbance at 625 nm.

#### 2.3.1 Photoinactivation Assay

PCPDTBT NPs were diluted to 17.0 mg L^–1^ in a 2 mL saline solution with *E. coli* inoculum (*ATCC* or *mcr-1*). After PCPDTBT NPs were added into the sample, the solution was placed in a shaker (Marconi, Brazil) and agitated at 120 rpm for 60 min. The sample was dispensed into a 96-well microplate containing 200 μL of the final solution and irradiated with an 806 nm infrared laser at 1.13 W for varying durations (0, 5, 10, and 30 min). At each time point, 10 μL was collected and serially diluted to a 1:32 dilution.

The bacterial plating was done using the Copacabana method^35^ with glass pearls in the Plate Count Agar (PCA) medium, with colony-forming units (CFUs) counted after 18 h of incubation at 37 °C. All experiments were performed in duplicate and repeated four times to determine the CFU values associated with the four nanoparticle concentrations tested in the irradiated and nonirradiated groups. Statistical analyses were carried out using Origin 8.5. CFU mL^–1^ values were subjected to variance analysis. Paired samples were compared using Student’s *t*-test at a 95% confidence level.

### 2.4 Morphological Assessment of Bacteria

Scanning electron microscopy (SEM; JEOL JSM-6380LV) was employed to analyze the morphological features of both irradiated and nonirradiated bacterial cells. Approximately 150 μL of the bacterial solution was initially placed in an Eppendorf microtube containing 1000 μL of glutaraldehyde and left to sit for 1 week after completing the photothermal experimental trial. Subsequently, 1000 μL of phosphate buffer solution (PBS) at pH 7.0 was added. Each sample was centrifuged at 1000 rpm for 5 min, and the supernatant was discarded. The centrifugation process was repeated three times with PBS, followed by a series of ethanol washes at concentrations of 25%, 50%, 60%, 70%, 80%, 90%, and finally absolute ethanol. The resulting precipitate was dissolved in absolute ethanol and then refrigerated. *E. coli* bacterial suspensions were immobilized on 1 cm × 1 cm glass substrates by drying overnight at room temperature. Next, a thin layer of gold was sputter-coated onto the substrates, which were then affixed to SEM holders by using conductive carbon tape. Micrographs were captured at a 15 kV accelerating voltage, 10 μm spot size, and 8 mm working distance.

## 3 Results and Discussion

### 3.1 Characterization of the Photothermal NPs

#### 3.1.1 Dynamic Light Scattering and Scanning Electron Microscopy

The high-resolution image of individual particles in a dry state shows particles with highly regular spherical shapes with a mean diameter of 120 ± 38 nm (Figure 1b,d). On the other hand, PCPDTBT NPs in solution exhibited a mean hydrodynamic diameter of 145 ± 16 nm that reflects the particles’ effective size, including their surrounding hydration layer and interactions with solvent molecules (Figure 1c).

The zeta potential is frequently used to characterize surface charge and quantitatively assess charge-induced colloidal stability in NP dispersions. The sign of the zeta potential indicates whether the particle surface is predominantly positive or negative. Typically, values exceeding +30 mV or falling below −30 mV suggest favorable electrostatic stability for NPs.^36,37^ The measured zeta potential for PCPDTBT NPs was −20.5 ± 0.7 mV, which falls short of the common threshold (±30 mV) required for optimal colloidal stability. The negative charge repels electrostatically, reducing the agglomeration. This interpretation is corroborated by the polydispersity index (PDI) of 0.48 ± 0.09, suggesting that PCPDTBT NPs are nearly monodispersed in the aqueous medium, as PDI values between 0.1 and 0.7 are considered indicative of monodispersity.^38^

The PCPDTBT NPs synthesized by Rohatgi et al. (2018) using the nanoprecipitation method exhibited a hydrodynamic diameter of approximately 70 nm and a zeta potential of −29.8 ± 1.6 in water.^39^

A study was carried out by Nagy-Simon and collaborators (2021) using PCPDTBT to prepare nanoparticles activatable by NIR via the nanoprecipitation method to perform fluorescence imaging and photodynamic and photothermal therapy applications; this work shows three concentrations of Pluronic and PCPDTBT, demonstrating that increasing the concentration also increases the size of NPs from 76 nm at the lowest concentration to 143 nm at the highest concentration.^40^

### 3.2 Optical and Thermal Characterization

Figure 2b shows broad absorption bands of the solution with PCPDTBT NPs (17.0 mg L^–1^) from the UV to the near-infrared range (350–900 nm). Abelha et al. (2019) previously studied PCPDTBT NPs for their potential as fluorescent agents in assessing near-infrared wavelengths in animal systems.^34,41^ The study also examined their cytotoxicity and hemocompatibility, particularly their compatibility with animal blood. The findings indicated that PCPDTBT NPs effectively produced near-infrared images, demonstrated low cytotoxicity, and exhibited high hemocompatibility, establishing them as reliable and biocompatible materials for various biomedical applications.^34^

**Figure 2:**
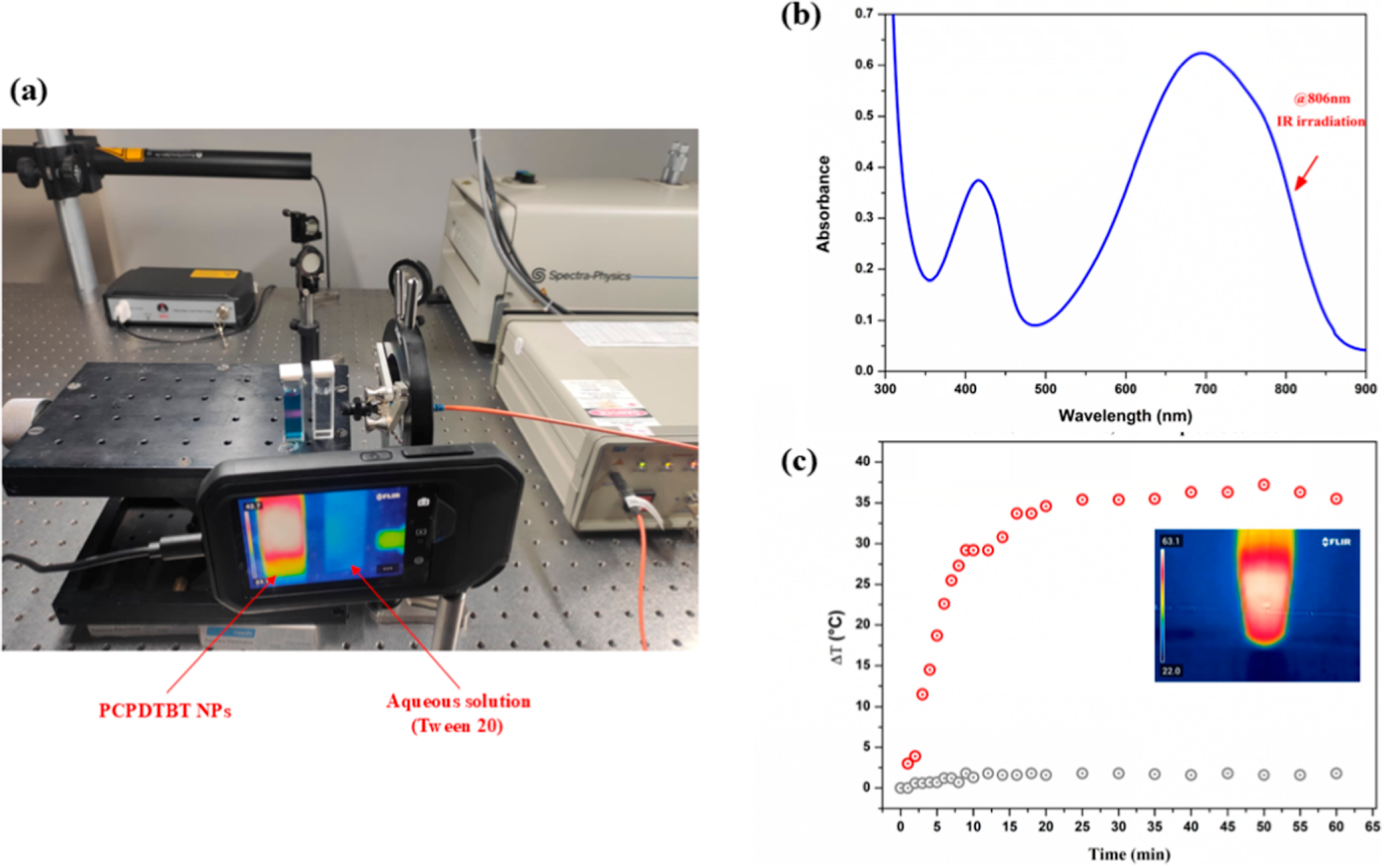
(a) Experimental setup for photothermal analysis; (b) absorption spectrum of PCPDTBT NPs (17.0 mg L^–1^). The arrow indicates the absorbance peak at 806 nm; (c) temperature as a function of time upon near-infrared irradiation (806 nm) for PCPDTBT nanoparticles (17.0 mg L^–1^) dispersed in aqueous solution and aqueous solution with Tween 20.

Several works use different matrices for the encapsulation of NPs and also associate them with other materials such as PCPDTBT; it was encapsulated with a layer of Pluronic F127 amphiphilic block and irradiated with a NIR laser at 785 nm, and the nanoparticles obtained present good thermal response and excellent photostability, leading to a photothermal conversion efficiency of 61%.^40^

### 3.3 Photothermal Response and Stability

It was possible to observe that the PCPDTBT NPs produced in Figures 3a and 3c require an adequate concentration and potency to obtain their peak efficiency, achieving a maximum temperature increase (Δ*T*) (°C) of 35–40 °C over time under infrared irradiation (806 nm) at different laser powers (0.22, 0.52, 0.81, and 1.13 W) at the concentration of 17.0 mg L^–1^ for PCPDTBT NPs and various concentrations of PCPDTBT NPs (0, 4.25, 8.50, and 17.0 mg L^–1^) a function of the time under infrared irradiation. Figures 3b and 3d also show the linear increase in temperature as a function of power and concentration, respectively.

**Figure 3:**
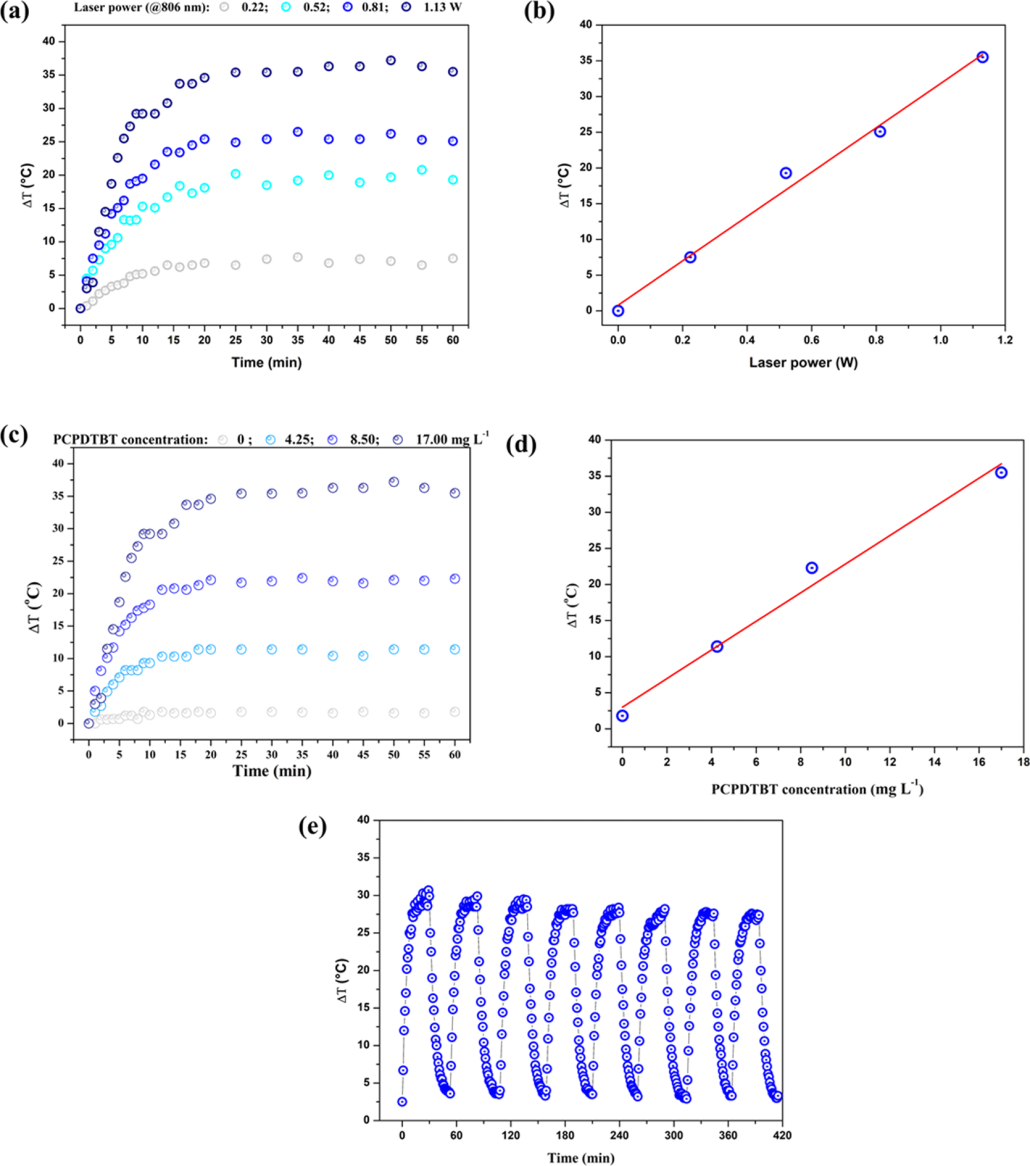
(a) Temperature of PCPDTBT NP solutions (17.0 mg L^–1^) as a function of time under 806 nm irradiation at laser powers (0.22, 0.52, 0.81, and 1.13 W); (b) solution temperature after 60 min of irradiation as a function of PCPDTBT NP concentration at 1.13 W (data from (a)); (c) temperature as a function of time of PCPDTBT NPs at different concentrations (0, 4.25, 8.50, and 17.0 mg L^–1^), irradiated at 806 nm irradiation at 1.13 W; (d) temperature of solutions after 60 min of irradiation extracted from data presented in (c); (e) cyclic photothermal heating of PCPDTBT NP solution (17.0 mg L^–1^) irradiated at 806 nm with 1.13 W over eight heating–cooling cycles.

Figure 3e demonstrates the thermal stability of PCPDTBT NPs at 17.0 mg L^–1^ even after 8 cycles of 60 min of irradiation at 1.13 W.

The PCPDTBT NPs have gained widespread application in the biomedical field since they have been used strictly as fluorescent markers^42,43^ and photoresponsive agents for heat generation, often in conjunction with other agents.^30^ The PCPDTBT-containing hybrid nanoparticles were also explored for the aPDI properties, acting as coagents for diagnostic imaging or photothermal therapy, used in association with other agents.^21,44,45^ Despite the existence of these works, this report presents, for the first time, the idea that nanoparticles composed solely of PCPDTBT can be utilized as a photothermal agent to inactivate multidrug-resistant bacteria.

### 3.4 Photoinactivation Assay and Bacterial Morphology

Figures 4a and 4b display representative images of *E. coli* colonies (strains ATCC and *mcr-1*, respectively) for the tested PCPDTBT NPs at the concentration of 17.0 mg L^–1^ as a function of the time (0, 5, 10, and 30 min) after being irradiated with an infrared laser (806 nm at 1.13 W).

**Figure 4:**
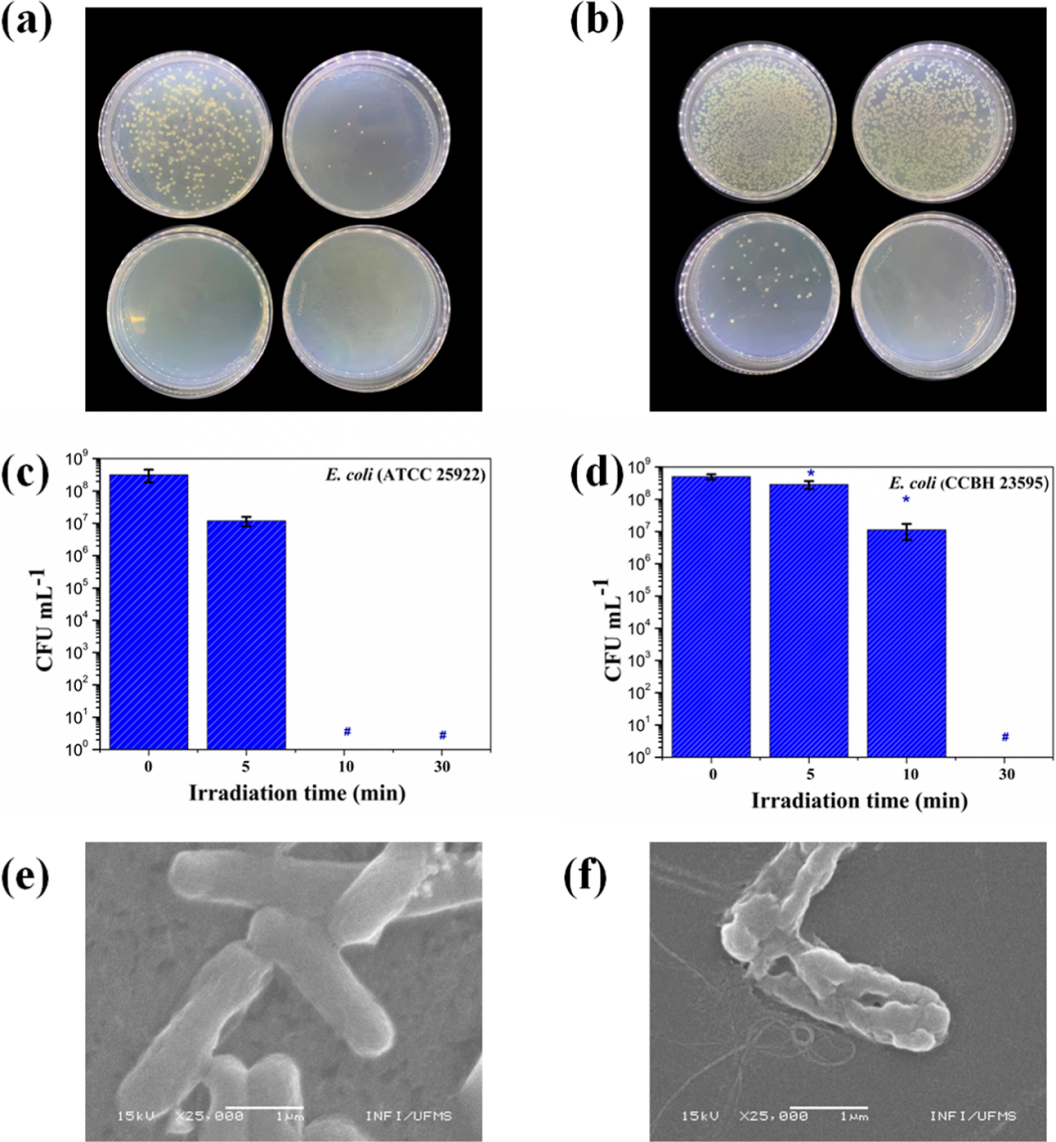
(a) Growth of *E. coli* (ATCC 25922) and (b) *mcr-1* positive *E. coli* (CCBH 23595) colonies in Petri dishes. Mean colony-forming unit values (CFU mL^–1^) for (c) *E. coli* (ATCC) and (d) *mcr-1* positive *E. coli* (CCBH 23593), as a function of 806 nm irradiation time (1.13 W), when exposed to PCPDTBT NPs (17.0 mg L^–1^). SEM images of *E. coli* exposed to PCPDTBT NPs (17.0 mg L^–1^): (e) nonirradiated and (f) after infrared irradiation (806 nm, 1.13 W) for 30 min. (*) indicates statistically significant difference at a 95% confidence level (*p* < 0.05) and (#) indicates the absence of bacterial colonies.

The mean CFU mL^–1^ values displayed in Figures 4c and 4d indicate the successful use of PCPDTBT NPs as photothermal agents against both strains of *E. coli* (ATCC and *mcr-1*) when exposed to laser irradiation (806 nm at 1.13 W) at the concentration of 17 mg L^–1^, resulting in a 97% reduction in CFU mL^–1^ against *E. coli* ATCC of the samples when comparing time 0 and 5 min, and the same reduction was observed for the *mcr-1* sample when comparing time 0 and 10 min. Additionally, it is crucial to emphasize that laser irradiation alone did not inactivate *mcr-1*-positive *E. coli* (data not shown), as expected, since no heat generation was detected in the absence of PCPDTBT NPs (Figure 2).

SEM images show the rod-shaped morphology of *E. coli* with an average size ranging from approximately 2 to 3 μm (Figure 4e). The micrographs depict the pristine, intact morphology of nonirradiated *E. coli* bacteria. In contrast, the process produced notable changes in the bacteria where photothermal therapy was applied, causing partial or complete damage to the bacterial cell wall upon exposure to an 806 nm laser for more than 10 min. In this case, the image was taken 30 min after irradiation (Figure 4f). The observed damage can induce bacterial lysis, effectively stopping the bacterial growth.

In a study conducted by Zhou et al. (2022), their nanoparticles, referred to as ALPBs, were produced using a double emulsion method with poly­(ethylene glycol) methyl ether-*block*-poly­(l-lactide-*co*-glycolide) (PLGA-PEG5000) as the matrix to encapsulate the dye TTDC, luminol, BNN6 (*N*,*N*′-di*sec*-butyl-*N*,*N*′-dinitroso-1,4-phenylenediamine), and PCPDTBT. Their bactericidal and photothermal properties, tested with 808 nm laser irradiation (1.0 W cm^–2^) for 10 min, effectively eradicated *S. aureus* and *E. coli* in vivo experiments, demonstrating their efficacy in synergistic photothermal-NO therapy, bearing in mind that we can assume from the graphs the reduction was more significant than 95% but less than 99% for the group irradiated and exposed to ALPBs.^26^

In the work by Zhou and collaborators, 10 min of irradiation was necessary to eradicate *E. coli*. Despite the different experimental conditions reported in this work, a 10 min of exposure to light was also used to eliminate *E. coli* ATCC. However, the *mcr-1* *E. coli* strain required a longer time for elimination (30 min). It remains unclear in the literature whether antimicrobial resistance to antibiotics confers increased tolerance to heat. A recent review concluded that there is no robust evidence indicating that antimicrobial-resistant bacteria exhibit greater heat resistance compared to nonresistant strains, except for some studies involving *Salmonella*, *S. typhimurium*, and MRSA.^46^ The literature consistently shows that thermal tolerance varies among bacterial species, serotypes, and strains, as well as across different substrates, regardless of whether the bacteria are antimicrobial-resistant (AMR) or not.^46^ Therefore, further studies are needed to elucidate the increased heating tolerance of *mcr-1* *E. coli* compared to that of *E. coli* ATCC.

## 4 Conclusion

In conclusion, PCPDTBT NPs are effective photothermal agents against the antibiotic-resistant *mcr-1* *E. coli* strain. The PCPDTBT NPs synthesized via nanoprecipitation exhibited robust photothermal properties characterized by efficient NIR light absorption and subsequent conversion into heat. This capability was validated through a consistent thermal performance over multiple irradiation cycles. Moreover, the photothermal efficacy of PCPDTBT NPs was confirmed through photoinactivation assays, revealing significant reductions in the number of viable bacterial counts after NIR laser treatment. The *mcr-1-*positive *E. coli* strain showed notable bactericidal effects, as illustrated by SEM images that revealed significant damage to bacterial morphology following photothermal treatment therapy. These findings highlight PCPDTBT NPs as promising candidates for combating antibiotic-resistant bacteria, offering a low-toxicity alternative to conventional antimicrobial treatments.

## Data Availability

The data are not publicly available due to intellectual property protection but are available from the corresponding author upon reasonable request.
